# Development of Ag-Doped ZnO Thin Films and Thermoluminescence (TLD) Characteristics for Radiation Technology

**DOI:** 10.3390/nano12173068

**Published:** 2022-09-03

**Authors:** Hammam Abdurabu Thabit, Norlaili A. Kabir, Abd Khamim Ismail, Shoroog Alraddadi, Abdullah Bafaqeer, Muneer Aziz Saleh

**Affiliations:** 1 Department of Physics, Faculty of Science, Universiti Teknologi Malaysia, UTM, Johor Bahru 81310, Malaysia; 2 School of Physics, Universiti Sains Malaysia, Pulau Pinang 11800, Malaysia; 3 Department of Physics, Umm AL-Qura University, Makkah 24382, Saudi Arabia; 4 Chemical Reaction Engineering Group (GREG), School of Chemical and Energy Engineering, Universiti Teknologi Malaysia, UTM, Johor Bahru 81310, Malaysia; 5 Office of Radiation Protection, Department of Health, Tumwater, WA 98501, USA

**Keywords:** dosimetry, Ag, ZnO, thermoluminescence, fading, linear response doses, sensitivity

## Abstract

This work examined the thermoluminescence dosimetry characteristics of Ag-doped ZnO thin films. The hydrothermal method was employed to synthesize Ag-doped ZnO thin films with variant molarity of Ag (0, 0.5, 1.0, 3.0, and 5.0 mol%). The structure, morphology, and optical characteristics were investigated using X-ray diffraction (XRD), scanning electron microscope (SEM), energy-dispersive X-ray spectroscopy (EDX), photoluminescence (PL), and UV–vis spectrophotometers. The thermoluminescence characteristics were examined by exposing the samples to X-ray radiation. It was obtained that the highest TL intensity for Ag-doped ZnO thin films appeared to correspond to 0.5 mol% of Ag, when the films were exposed to X-ray radiation. The results further showed that the glow curve has a single peak at 240–325 °C, with its maximum at 270 °C, which corresponded to the heating rate of 5 °C/s. The results of the annealing procedures showed the best TL response was found at 400 °C and 30 min. The dose–response revealed a good linear up to 4 Gy. The proposed sensitivity was 1.8 times higher than the TLD 100 chips. The thermal fading was recorded at 8% for 1 Gy and 20% for 4 Gy in the first hour. After 45 days of irradiation, the signal loss was recorded at 32% and 40% for the cases of 1 Gy and 4 Gy, respectively. The obtained optical fading results confirmed that all samples’ stored signals were affected by the exposure to sunlight, which decreased up to 70% after 6 h. This new dosimeter exhibits good properties for radiation measurement, given its overgrowth (in terms of the glow curve) within 30 s (similar to the TLD 100 case), simple annealing procedure, and high sensitivity (two times that of the TLD 100).

## 1. Introduction

Thermoluminescence dosimeter, well-known as TLD, is a device that can keep radiation energy for a specific time and then read it out after being induced by heating. The process behind this device depends on the emission of light. TLD is a tool for measuring radiation doses in the clinical, radiotherapy, environmental, irradiated food, industrial, and quality assurance fields [1,2,3,4,5,6,7,8]. TLD remains the most powerful method to measure radiation doses due to its reliability, non-intricacy, and portability [1,3,9,10,11]. The thermoluminescence (TL) mechanism depends on impurities defects in the crystal structure, which increment the capacity of the materials to store radiation energy [6,11,12,13].

In recent years, many studies have been performed to establish novel high-performance TLDs that show a linear response at a wide range of doses, since most dosimeters exhibit nonlinear responses in a wide range of doses. TLD materials are microcrystalline powders or chips that can disperse light, where the near-surface light produced can approach the photon detector more than the light from within its depth. This result depends on the dosimeter’s ability to keep light emitting through it. Most researchers focus on block form dosimeters such as pellets, chips, and discs [4,14,15,16,17]. In contrast, a few researchers have used limited efforts to investigate nanocomposites with a creative design of 2D thin films to serve as TL materials [18]. We see that it is of the utmost importance to consider modifying the dimensions of the dosimeter, to ensure that it is compatible with various applications and the critical regions for radiation measurements.

The development of thin films with dosimetric characteristics is of great importance in calculating low penetrating radiation doses, including in the study of dose distribution at interfaces. However, knowing the dose in the particular region and beyond clinics are significant. These are essential assets of knowledge to prevent unnecessary issues in the skin and treat the lymphatic system at a depth of 0.5 mm. According to the literature review, several attempts have been made to design two dimensional (2D) dosimeters [19,20,21,22]. Additionally, the 2D design of the dosimeter with a high resolution is essential in modern radiotherapy, for modalities where steep gradients of dose distributions occur. Moreover, overcoming the problems associated with measuring depth dosage distributions is desirable [14,23,24].

Hence, Zinc oxide, ZnO, is an excellent advantage in improving efficient TL phosphor tailored to be used in dosimetry applications, due to its wide bandgap of 3.37 eV, exciton binding energy of 60 MeV, and high transparency of 90% in the visible region [23]. Based on the characterization of ZnO, the surface area to volume ratio increases with the decrease in the nano-range of grain sizes, which changes the optical properties such as transmittance. Moreover, ZnO has inherent structures with different morphologies, i.e., nanoparticles, nanowires, and nanorods [24]. However, ZnO has weaknesses, such as a high electron-hole recombination rate. To address this limitation, doping with foreign atoms is crucial for modifying the characteristics and proposed uses of semiconductor nanocrystals. Adding impurities to ZnO will change the emission luminescence, as it creates defects in materials and increases the charge carriers. The results of ZnO doped by transition metals showed ZnO as a promising composite material in dosimetry, based on the TL glow curves, per the most recent literature review indicated in our previous work [25]. The introduction of Ag to ZnO caused the substitute of Ag^2+^ in the Zn^2+^ lattice, which increased the oxygen vacancies due to variation in the ionic radius between Ag (1.15 Å) and ZnO (.72 Å). These vacancies act as the sub-bandgap donor sites, producing traps; these sub-bandgap act as traps for the electrons during irradiation. Later by stimulating the nanocomposite, the trapped electrons tend to relax in the recombination center. A typical recombination center may be created by dislocating a negative ion that works as an electron trap; if this trap is shallow may be released by thermal vibrations of the lattice. On the other side, if the trap is deep (high activation energy), the electrons will recombine with the holes’ trap at the recombination center, giving rise to light emission (TL).

Therefore, adding selective elements to ZnO offers a vital way to enhance and control optical and luminescence properties. According to the theory of valence control in oxide semiconductors, the Debye length (*L_D_*) of ZnO is reduced when it is doped with acceptor elements such as Au, Cu, and Ag; Ag can promote the separation of spatially generated charge carriers. Furthermore, the unique interface interactions between Ag metal and ZnO may be related to the presence of the Schottky barrier, which promotes charge carrier separation. For instance, Huang observed that when Ag (NPs) is added to ZnO, electrons concentrate in ZnO along the Ag–ZnO interface until the electron-rich islands link. This difference in electron transport at Ag–ZnO is caused by the fact that the work function of ZnO (4.62 eV) is greater than that of Ag (4.24 eV), increasing electric conductivity up to 1000 times.

Furthermore, when the Ag content in the ZnO matrix increases, the electron concentration rises to 2.4 10^20^/cm, causing the electron accumulation zones to overlap and forming a percolation channel for electron transport, without reducing electron mobility [26]. Similarly, Corro and their research group reported that adding Ag to pure ZnO increased the number of electrons in the conduction band (CB) of ZnO. This is caused by the interfacial electronic interactions between the metals and the ZnO. These impurities may increase the probability of localized electrons being trapped in de-traps (close to conduction band CB); this might occur because the electron transfer that emerged from Ag to ZnO caused the shifted absorption to a higher wavelength [27]. Saboor’s findings demonstrated that the Ag-doping concentration significantly impacted the morphology, structure, and intrinsic defects of ZnO nanorods. As the modifier shifts the conduction band and Fermi level of the ZnO nanorods, it creates vacancies and forms ionic bonds with the oxygen atom rather than covalent bonds [28]. Likewise, incorporating silver into ZnO creates surface defects, which act as effective charge carrier traps to reduce the recombination rate of the photogenerated charge carriers. Thus, the addition of Ag to ZnO caused an increase in oxygen vacancies sites, due to the electron sensitization effect of Ag; as a result, an improvement in TL intensity can be obtained [29]. However, scholars are still making intensive efforts to improve the TL properties of these phosphors, either by preparing them in various ways, doping them with different impurities, or introducing new matrices, with ZnO being one of these new host materials. Although many researchers have been dedicated to developing a ZnO-based dosimeter, to our knowledge, no study has been reported that covers all of the features [30,31,32,33,34]. This study comprehensively investigated the dosimeter characteristics of Ag-doped ZnO thin films grown via the hydrothermal method.

## 2. Experimental Section

### 2.1. Materials

All of the compounds chosen for this research were conducted without additional purification. Zinc acetate dihydrate (Zn(CH_3_COO)_2_·2H_2_O) (BHD, Poole, UK), silver nitrate AgNO_3_ (Sigma, Ronkonkoma, NY, USA), hexamethylenetetramine (C_6_H_12_N) (HMTA, Merck, Darmstadt, Germany). The ammonia (NH_3_) and ionized water were available in the laboratory of School of Physics Universiti Sains Malaysia.

### 2.2. Preparation of Ag-Doped ZnO Thin Films and Measurements

The simple hydrothermal method was employed to synthesize Ag-doped ZnO thin film growth on the glass. The solution was prepared by mixing 0.1 mol of Zinc acetate dihydrate and 0.1 mol of hexamethylenetetramine in 100 mL of ionized water. After that, the mixture was vigorously stirred for half hour. AgNO_3_ (0, 0.5, 1.0, 3.0, and 5.0 wt%) was introduced to the prepared solution and continuously stirred for 1 h to obtain a homogeneous solution. Then, NH_3_ was added dropwise into the solution until the transparent solution was acquired. The solution was then poured into 150 mL Teflon autoclave, with the immersion of the glass substrate (slab glass that deposited the thin films on it), before it was closed tightly and heated at 180 °C in the furnace for 12 h, after which it was cooled to ambient temperature. The thin film was rinsed several times using deionized water. Finally, the sample was annealed at 400 °C for 1 h in the furnace for impurity removal. This procedure was performed several times for different concentrations of Ag, as previously mentioned.

Meanwhile, the structure analysis of pure ZnO and Ag-doped ZnO thin films was measured by X-ray diffraction (XRD) (Malvern PANalytical, Malvern, UK) and equipped with a Cu-K emission wavelength at (0.154 nm), for the range of 2θ at 20° ≤ 2 θ ≤ 80°. Besides that, the morphology of the thin films was studied by scanning electron microscope (SEM) (JSM-6460LV SEM JEOL Ltd, Tokyo, Japan). The nanocomposite of the thin films was depicted via energy-dispersive X-ray (EDX) (Tokyo, Japan) analysis. In addition, photoluminescence (PL) (Jobin Yvon HR 800 UV Jobin Yvon, Kyoto, Japan) was investigated using a laser with a power of 0.18 mW, and the excitation source’s wavelength was 325 nm. Ultraviolet visible (UV–vis) spectrophotometry (Model Cary 5000 UV-VIS-NIR Agilent Technologies, Santa Clara, CA, USA) was applied to measure the transmittance.

### 2.3. Thermoluminescence Measurments

**Figure 1:**
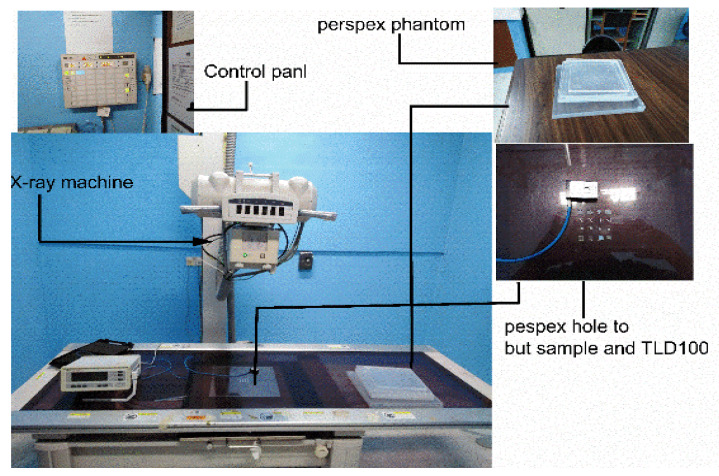
The schematic diagram of the experimental set-up for X-ray irradiation dose using Toshiba KXO-50S X-ray.

In this study, the process of preparing the samples was carefully considered; the weighing by difference technique was applied to measure the mass of the thin films. After that, the thin film was cut into 5 × 5 mm^2^. The samples were then labeled and placed in opaque containers. Before irradiating the samples, the annealing dosimeter procedure was first conducted to remove any traps. Annealing treatment is a method used to eliminate the residual signal, which may cause unwanted background readings in this work. The samples were kept under specific circumstances, to shield them against external physical and environmental effects such as dust and cleaning. Figure 1 shows the setting of the samples for irradiation using an X-ray machine (Toshiba KXO-50S) (Toshiba Medical Equipment, Tokyo, Japan) with its control panel setting, as mentioned in our previous study but with a slight modification, where the SSD and the field size were set at 80 cm and 10 × 10 cm^2^, respectively [35]. In the present work, the temperature–time profile was preheated at 50 °C, and the maximum temperature was 400 °C. The heating rate was carried out within the intervals of (1, 3, 5, 7, and 10 °C/s) for all three compositions, where the samples were irradiated by X-ray radiation, and each data point is an average of three samples to estimate the average TL intensity and standard deviation. Win REMS application software (USA) for TLD readers involved two phases of preheating.

There are two steps in the readout (acquisition phase): collecting light emitted during the heating process and converting the light into an integrated value (display glow curve). In order to measure the percentage depth doses (PDD), 10 Perspex phantom slides were used, where the thickness of each slide was 1 cm. HARSHAW TLD Model 3500 (Thermo Fisher, Waltham, MA, USA) was utilized. Initially, the reader was warmed for 30 min, and nitrogen gas was turned on at a flow rate of 3.0 to 4.0 psi. The planchet’s nitrogen flow improves the accuracy of low exposure reading and extends the planchet life by eliminating the oxygen in the planchet area. The time temperature profile (TTP) included the heating cycle parameters and was set in the Win REMS software. The TL charge was collected from each reading cycle in 200 data points.

## 3. Results and Discussion

### 3.1. Structural, Morphological, and Chemical Composition Investigations

**Figure 2:**
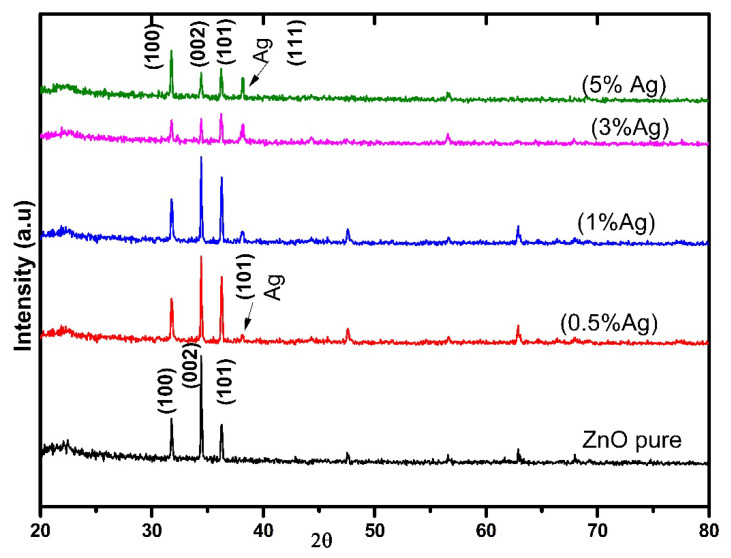
The X-ray diffraction pattern of undoped ZnO and Ag-doped ZnO thin films.

Figure 2 displays the structure and crystallinity of undoped ZnO and Ag-doped ZnO thin films using XRD (20° ≤ 2 *θ* ≤ 80°). The results showed polycrystalline with a hexagonal wurtzite structure for the Ag-doped sample [36]. The diffraction peaks corresponding to ZnO and Ag appeared to agree with the standard JCPDS data card 01-089-0511 and 03-065-2871, respectively. The peaks for all samples indicate that Ag-doped ZnO thin films have grown successfully. The peaks of 100, 002, and 101 planes correspond to 2 *θ* = 31.77°, 34.37°, and 36.35° of ZnO, respectively. The peak at (002) orientation is the highest, indicating the growth direction and c-axis orientation. In the meantime, the diffraction peak of 111 at 2 θ = 38.22° is consistent with Ag for Ag-doped ZnO thin films. No evidence of impurity peaks was observed from the XRD data [37].

The results showed that the diffraction peaks connected to ZnO decreased along with the Ag dopant increment, due to the disorder formed by the Ag ions in the ZnO lattice structure; moreover, the dopant’s chemical reactivity plays a role in crystal growth dynamics. As a result, the added Ag atoms cause a deformation in the crystalline structure of ZnO [38]. However, this deformity may be ascribed to Ag^+^ having a considerably larger ionic radius (1.22 Å) than Zn^+^ 0.72 Å, leading to the segregation of Ag atoms to the grain boundaries of ZnO crystal, indicating Ag cluster formation that appeared as separate Ag peaks. Consequently, a metallic phase of silver was produced on the surface of ZnO [28,39].

The crystallite size was estimated using Scherrer’s formula and was tabulated in Table 1:

(1) D=0.89λ/βcosθ

As seen in Table 1, the TL intensity increases as the crystallite size decreases. The change of TL is consistent with that of the surface fluorescence, which increases as the surface area to volume ratio increases, as we mentioned in the introduction. When the nanocomposite is exposed to radiation, the electron and hole will be created and trapped in these metastable states. Hence, it is obvious that the TL of the nanoparticles is proportional to the surface defects. The surface-to-volume ratio increases when the size decreases, and the particles go to more easily obtainable carriers, which increase the holes and electrons for TL emission. There is still much to learn about the aspects of the upconversion of luminescence in these nanomaterials [40,41,42,43]. However, by increasing the dopant, the intensity decreased. Researchers ascribed the decrease in luminescence intensities to the quenching effect as an increasing dopant, which is consistent with this study [44,45].

**Figure 3:**
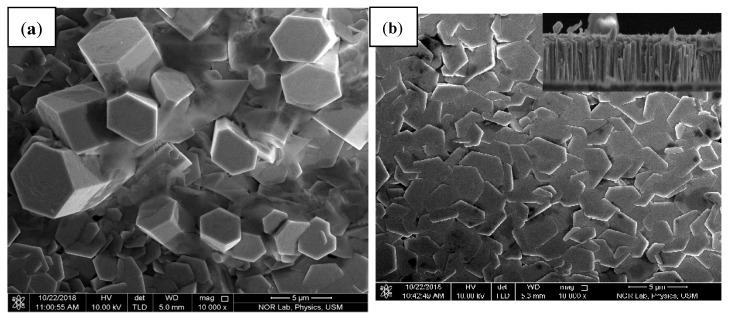

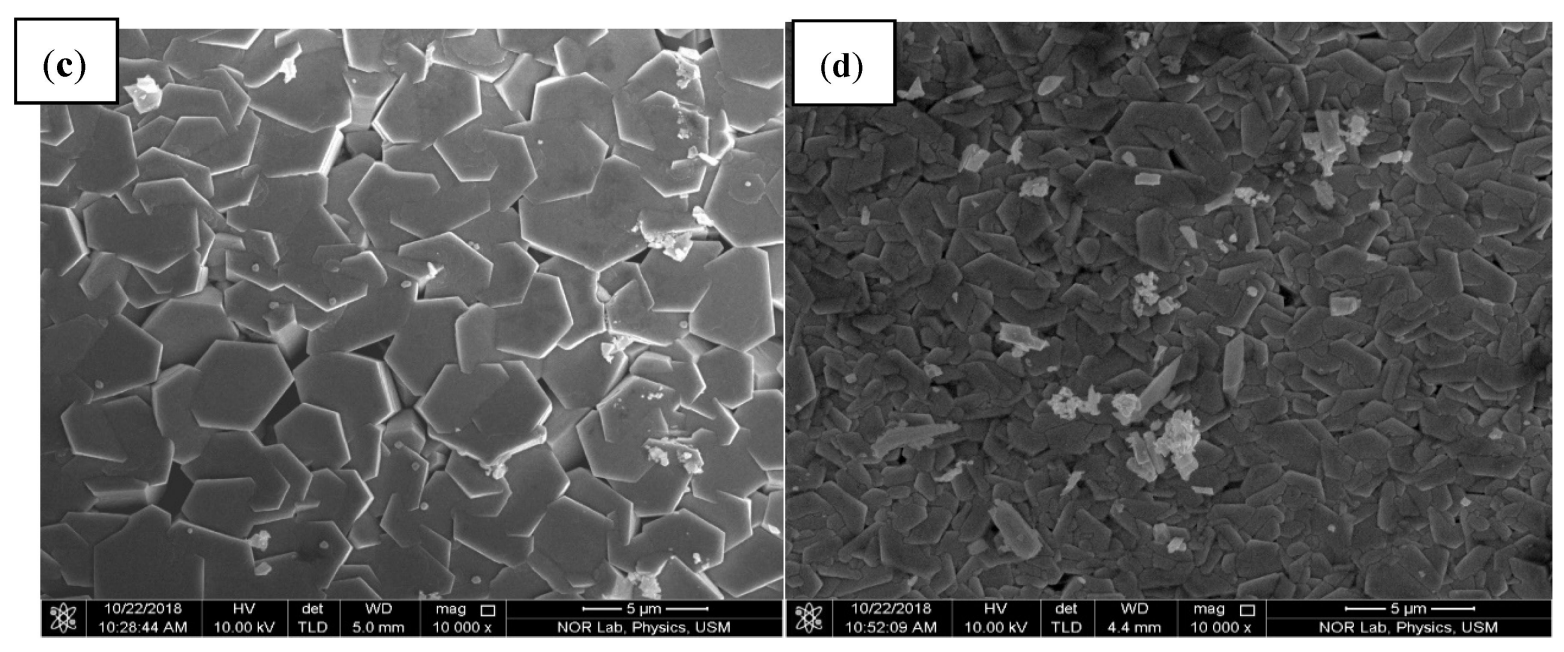
FE-SEM images of the (**a**) pure ZnO and silver dopant at (**b**) 0.5%; (**c**) 1%; and (**d**) 3%, respectively.

Figure 3’s SEM images show the surface morphology of pure ZnO and Ag-doped ZnO thin films. One-dimension nanostructure hexagonal shapes were successfully synthesized through the hydrothermal method. The effect of the amount of Ag on the morphology of ZnO nanorods is observed. Based on the obtained results, the nanorods’ length is 5 μm, as confirmed from the cross section (image inset of Figure 3b). The surface morphology of Ag-doped ZnO became deformed and flattened by increasing the Ag dopant. The morphologies of thin films are affected by adding Ag^+^, which may be replaced with Zn^2+^. Further increase in impurities caused the agglomeration of Ag atoms at the grain boundary of ZnO crystal, suggesting the creation of Ag clusters that appeared as separate Ag atoms, as can be seen in Figure 3c,d [46].

**Figure 4:**
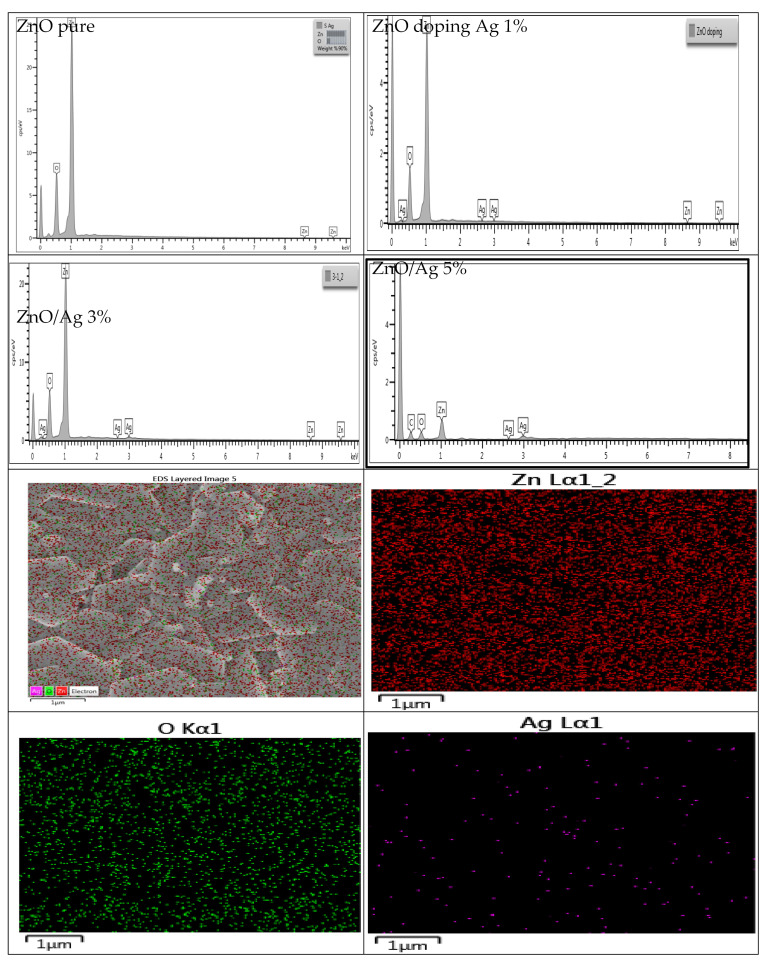
The EDX spectrum for undoped and Ag-doped ZnO thin films with typical mapping images.

Energy-dispersive X-ray (EDX) spectroscopy was used to evaluate the chemical compositions of pristine ZnO and Ag-doped nanorods. The results showed that the sample comprises Zn and O elements; no additional elemental peaks were seen in the pristine ZnO analysis. Ag impurities have also been fully incorporated into the ZnO lattice. Figure 4 showed the elemental synthesis of ZnO and Ag-doped ZnO thin films via EDX and revealed a good agreement with the experimental composite [47]. The mapping images exhibited a uniform and homogeneous distribution of Ag^+^ ions in ZnO.

### 3.2. Optical Properties Studies

**Figure 5:**
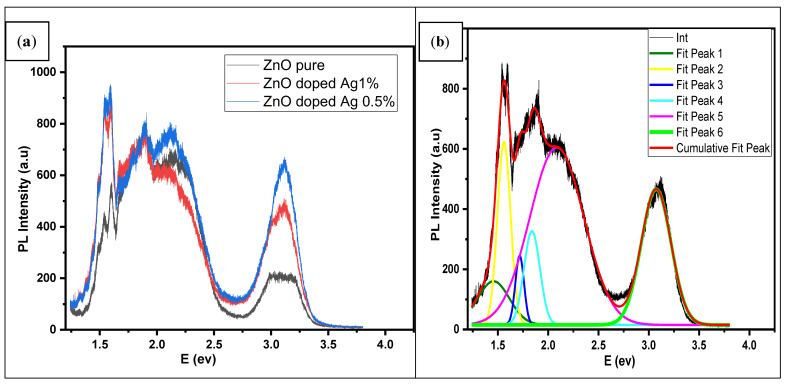
(**a**) Room temperature PL spectra of pure ZnO and Ag-doped ZnO 1%, 0.5% mol thin films, and (**b**) Gaussian deconvolution of Ag-doped ZnO thin film.

The PL spectra of undoped ZnO and Ag-doped ZnO thin films at room temperature were recorded. Figure 5a shows the PL spectra of pure ZnO and Ag-doped ZnO as-synthesized by hydrothermal deposition at room temperature, after annealing at 400 °C for 2 h. The PL study was conducted at room temperature with an excitation wavelength of 325 nm xenon-lamp Laser. The obtained results revealed two peaks that included the near bandgap (NBE) (3.26 eV), due to the collision process of excitons. The other highest intensity peak in the visible region is defined as deep level emission (DLE) with a wide range (2.25 to 1.46 eV); this was mainly attributed to the electron-hole recombination, which gave rise to the surface and intrinsic defects in the crystalline structure lattice during the growth. In addition, the DLE is the result of various developmental defects, such as zinc interstitials (Zn_i_), interstitial oxygen (O_i_), zinc vacancies (V_Zn_), and oxygen vacancies. These defects cause the DLE (V_O_), and the red band at 1.6 *E_v_* was observed due to the interface between O_i_ and V_Zn_ emissions [48,49,50,51].

For the Ag-doped ZnO thin film, there is an increase in the intensity of NBE without shifting the peak’s position. The enhancement in UV intensity after annealing may be attributed to excitonic recombination; we can explain it as due to the implanted Ag atoms; thermal annealing provides energy to occupy Zn atom sites in the lattice of ZnO. When no Ag atoms are stuck in non-equilibrium positions, the occupying probability could increase the temperature and gradually become steady at a specific value. The UV light in ZnO crystal can excite photocarriers [51,52]. However, the optimized intensity was 0.5 mol%, due to the electron-hole recombination as an electron transporting layer and the oxygen vacancies mechanism. With the increase in Ag molarity beyond 0.5% of the Ag amount, the PL intensity tended to reduce, which may be due to the surface plasmon resonance (SPR) of Ag, as reported in a prior study by [53].

The cause of the red region is still controversial. Due to the diverse array and complexity of defects existing in ZnO, some authors have attributed it to ZnO structure defects. In contrast, some other authors attributed it to an excess of oxygen impurities [54]. To understand the characteristics of the broad visible emission band, the deconvolution of the components band was applied via Gaussian fitting, see Figure 5b. The deconvolution peak at 2.10 (eV) may be ascribed to the V_Zn_–V_O_ vacancies that other scholars assigned to positively charged oxygen vacancy V_O++_ [54,55,56,57]. Moreover, we observed other peaks at 1.84 (eV) and 1.7 (eV) assigned as the yellow and orange emissions in ZnO, respectively, attributed to neutral V_O_ and interstitial zinc atoms Zn_i_ [58,59,60,61]. The emission bands in the 1.7 (eV), 1.56 (eV), and 1.46 (eV) were caused by different types of defects, such as interstitial zinc, zinc vacancy, oxygen vacancy, and interstitial oxygen [56,62,63,64]. The emission bands have been improved by oxygen heat treatment and Ag-doping, suggesting that this emission band is due to Ag–O clusters [18].

**Figure 6:**
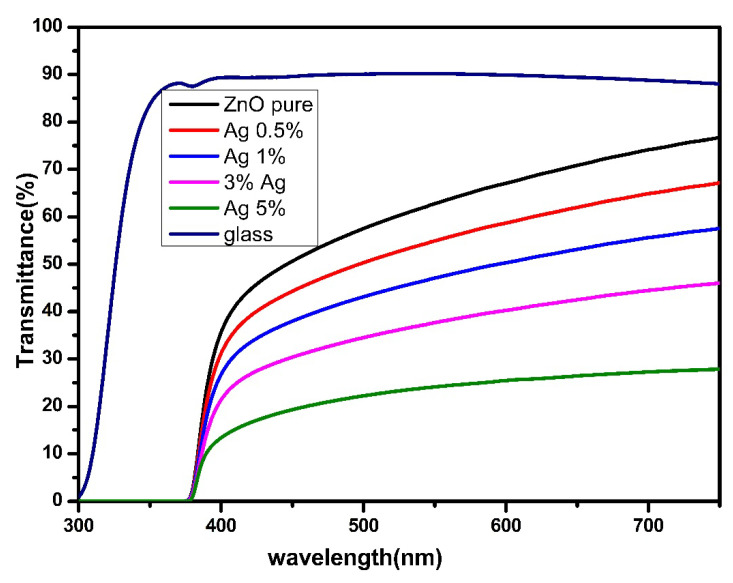
Optical transmittance for undoped ZnO and Ag-doped ZnO thin films.

Figure 6 shows the transmittance spectra of ZnO and Ag-doped ZnO over 200 to 800 nm. The results revealed that the transmittance decreased with the increment of Ag, which may be ascribed to the scattering of photons by crystal defects formed by the Ag dopant, agglomeration density, and adsorption of free carriers [65].

**Figure 7:**
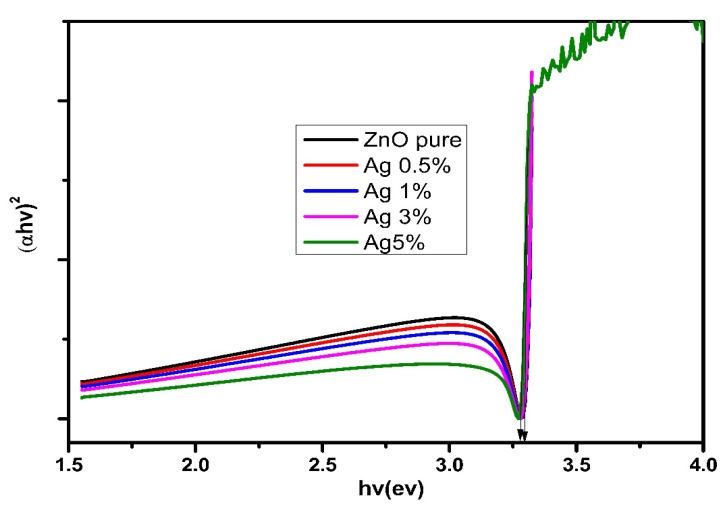
The bandgap for undoped ZnO and Ag-doped ZnO thin films.

Figure 7 shows the bandgap (*E_g_*) of the samples. From the above transmittance, the absorption coefficient (*α*) was determined based on the following equation:

*α = −LnT/d* (2)

where *T* is the transmittance, and *d* is the thickness of the thin films. Meanwhile, the bandgap can be measured by extrapolating the linear portion curve of (αhν)^2^ versus the photon energy (*hν*), according to the following equation:

*αhv* = (*hv* − *Eg*)^m^ (3)

where *E_g_* is the gap energy; *hν* is the energy of the photon; *α* is the calculated absorption coefficient from the raw transmittance data; and *m* = 1/2 for the plotted direct transition bandgap (*αhv*)^2^ vis photon energy (*hv*).

The energy gap for ZnO recorded 3.2 eV, slightly decreasing with the addition of Ag. The decrease in *E_g_* with the increase in Ag may signify Ag^+^ was substituting for Zn^+^ in the lattice [66]. The production of oxygen vacancies plays a vital role in reducing the bandgap, as they serve as trap centers that minimize the recombination of charge carriers by capturing the electrons. Furthermore, the addition of Ag may cause defects in the bandgap, which can broaden the spectrum and promote emission in the visible range, leading to improved luminescence characteristics of Ag-doped ZnO nanocomposites [67].

### 3.3. Dosimetry Characteristics

#### 3.3.1. Sample Optimization

**Figure 8:**
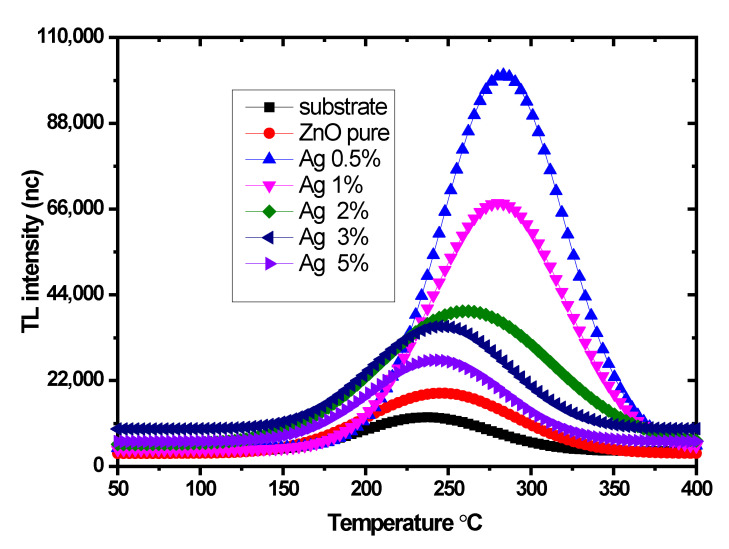
The glow curves of Ag-doped ZnO thin films with different concentrations of Ag percentage.

The samples were exposed to 3 Gy of X-ray radiation. Figure 8 displays the glow curve of the dosimetric peak for ZnO and Ag-doped ZnO thin films. The acquired glow curve exhibited a single peak sited at 240–270 °C with 5 °C/s of the heating rate. The results showed that the highest intensity of the thermoluminescence corresponded to a 0.5 mol% Ag-doped ZnO sample at 270 °C. This temperature peak is a desirable site for the dosimetry application because, if located at a low temperature, it will cause an increase in the fading and a loss of the signal, similar to if the dosimetric peak is situated at a high temperature, which leads to an interface with black body radiation as reported [25,68]. As the concentration of Ag decreased, the TL intensity appeared to increase, with a noticeable shift toward higher temperature.

The emission of TL intensity for Ag-doped ZnO increased when the dopant ratio reached 0.5%. Beyond this concentration, the Ag atoms became agglomerated into metallic Ag clusters, reducing the surface defects and, thus, quenching the TL intensity [69,70].

Another fact is that Ag-doped ZnO does not create a new TL peak but increases the trap center, increasing TL intensity. For more explanation of the TL mechanism of Ag-doped ZnO, when the samples were irradiated (the photon energy of X-ray was more significant than the bandgap of ZnO), the electrons in the valence band of this nanocomposite excited to the conduction band $\left(e^{-}\right)$ simultaneously produce an equal number of holes (*h^+^*) in the valence band. These electrons and holes will relax at the tarps created by the oxygen vacancies and Ag+ ions, preventing the immediate recombination of (*e*^−^ − *h*^+^) pairs. Consequently, the electrons and holes will stay trapped for a long time, depending on the lifetime of the levels trapped and the ambient temperature; later, when stimulated, the samples via the temperature, electrons, and holes will recombine at the recombination center, and emission photons will occur. The optimized sample of 0.5%Ag-doped ZnO was chosen for further investigations.

$$\mathrm{ZnO}/\mathrm{Ag}++\mathrm{radiation}\;\to \;\mathrm{ZnO}/\mathrm{Ag}\;(e_{cb}^{-}+h_{vb}^{+})$$

$$(e_{cb}^{-}+h_{vb}^{+})\;\to \;\overset{recombined\;at\;LM\;center}{\to }\;\mathrm{emission}\;\mathrm{light}$$

#### 3.3.2. Heating Rate

**Figure 9:**
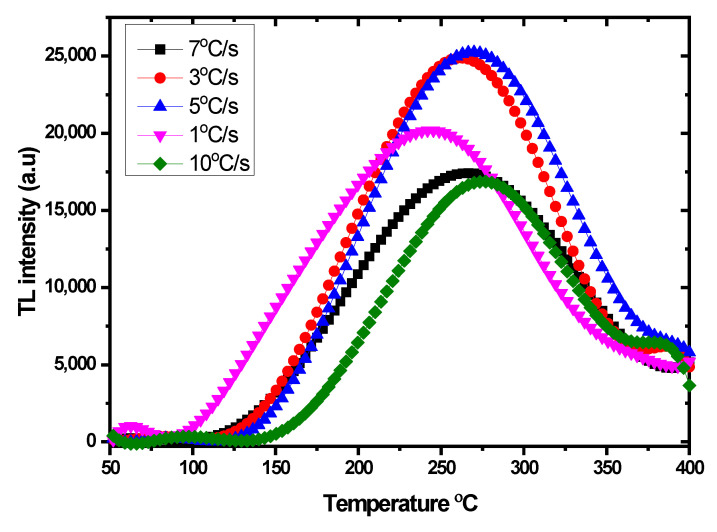
Glow curve of Ag-doped ZnO thin films as a function of heating rate.

**Figure 10:**
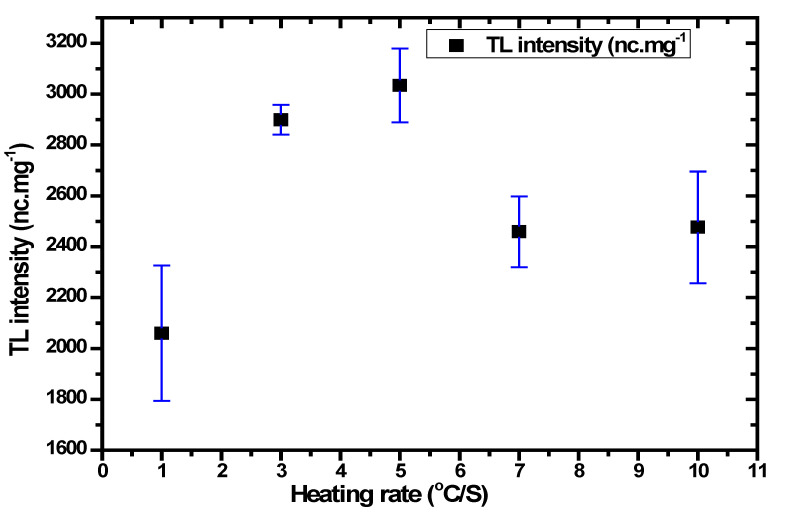
TL response of Ag-doped ZnO thin films as a function of heating rate.

The process of determining the heating rate is crucial in order to acquire the optimal recombination and sensitivity [71]. Figure 9 depicts the glow curve with a different heating rate of the optimum sample. For this study, each data value identified an average of three dosimeters samples to estimate the average TL intensity and a standard deviation. The prepared samples were automatically heated at 50 °C by the TLD reader before recording the glow curve, and the temperature was gradually incremented at the heating rate of 1 °C/s up to 10 °C/s. Ideally, the optimum heating rate should achieve the highest TL intensity with the lowest standard deviation [72]. As shown in Figure 10, the optimum heating rate corresponding with the highest TL intensity is set at 5 °C/s. It was observed that the intensity of the glow curve with the different heating rates of the Ag-doped ZnO film decreased when increasing the heating rate. The glow curve is connected to trap levels in the bandgap between the conduction bands and the valence bands of material at varying depths. These trap levels are distinguished by kinetic parameters such as activation energy, live time, and frequency factor. Besides, the reduction in intensity with an increase in heating rate can be attributed to the association between the time taken to de-trap the electrons trapped and the number of electrons de-trapped due to thermal stimulation [73].

#### 3.3.3. Annealing Procedures

Annealing treatment is a method used to eliminate the residual signal of radiations, which may cause unwanted background readings; this process is essential to yielding a precise TLD outcome [74] The annealing procedure in the current study was carried out using different temperature values (100–400 °C) at a specific annealing time (1 h). In this investigation, each data value indicated an average of three dosimeter samples. Following that, the samples were exposed to 3 Gy of X-ray radiation.

**Figure 11:**
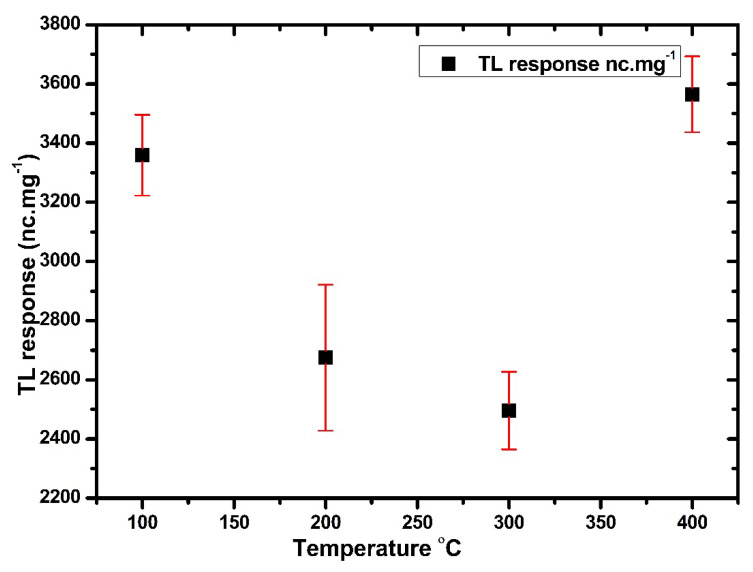
The TL response of Ag-doped ZnO thin films with different annealing temperatures.

**Figure 12:**
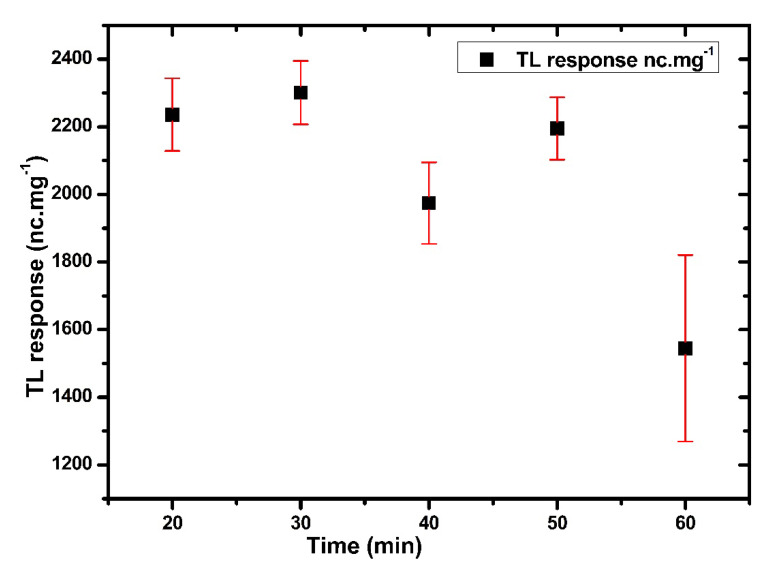
The TL response of Ag-doped ZnO thin films with different annealing times.

Figure 11 displays the best TL response with a minimum standard deviation of the Ag-doped ZnO (0.5 mol%) at 400 °C. The samples were subjected to an annealing temperature at 400 °C with different annealing times (from 20 to 60 min), before being exposed to 3 Gy of X-ray radiation. As shown in Figure 12, the optimum TL response and low standard deviation were found at 30 min. These annealing processes demonstrated that all traps had been evacuated and restored the thermodynamic defect equilibrium before the irradiation [75]

#### 3.3.4. Dose-Response

**Figure 13:**
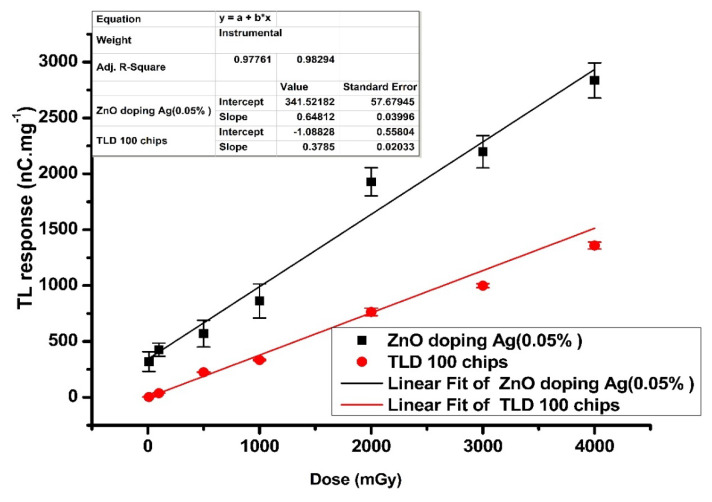
The linearity of response dose irradiated with X-ray (0.1–4) Gy.

The study investigated the TL response for the Ag-doped ZnO thin films. Each data point involved three samples; the average and standard deviations were taken. Figure 13 shows the linear response doses (0.1–4 Gy) for Ag-doped ZnO thin films. The study revealed an excellent linear response and correlation coefficients of 0.97 and 0.996 for Ag-doped ZnO thin films and TLD 100 chips, respectively. The results indicated the distribution of deep traps in Ag-doped (0.5 mol%) ZnO thin films located at different levels [76]. The Ag-doped ZnO thin films and TLD 100 chips were irradiated under the same condition for comparison (to the material host). The response of TLD 100 chips appeared linear within the range (0.1–4 Gy), as expected. This finding confirmed that the phosphorus established deep traps within the nanocomposites lattice, which is proportional to the various doses [77].

#### 3.3.5. Sensitivity

One of the essential characteristics of TLD properties is dosimeter sensitivity TL. Sensitivity is defined as the TL intensity per unit mass of the thin film and per unit dose of X-ray radiation (TL.mGy^−1^.mg^−1^), as shown in Equation (4).

(4) $$S\left(D\right)=TL/m.D$$

where *TL* is the intensity (expressed in nC), and *m.D* is the dose (expressed in mGy).

Relative sensitivity, another term linked to the dosimeter material sensitivity, is often compared to the standard (TLD 100). The following equation expresses relative sensitivity [78].

(5) $$R\left(D\right)=\;S{\left(D\right)}_{material}/S{\left(D\right)}_{TLD\;100}\;$$

The sensitivity could be obtained based on the slope of the graph of response doses (linearity) [79]. The sensitivity of 0.5 mol% Ag-doped ZnO appeared to be approximately two times that of the TLD 100 chips, which agreed with the past reported studies.

#### 3.3.6. Reproducibility

**Figure 14:**
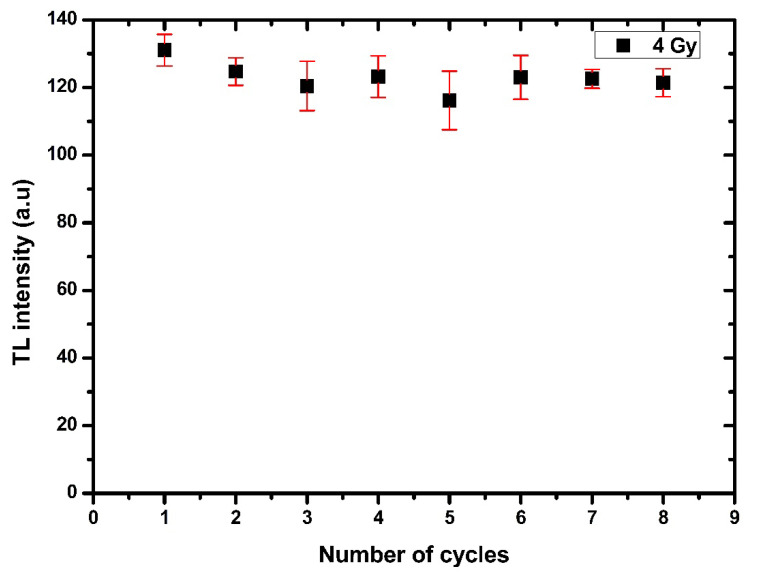
Reproducibility of Ag-doped ZnO thin films irradiated 4 Gy with X-ray.

Reproducibility is another significant aspect of TL material that should stay stable even after repeated usage. The samples were irradiated to 4 Gy and stored for 24 h at room temperature. The numerous cycles of TL reading were recorded. As a result, Figure 14 shows no considerable difference in the results after eight cycles and no changes in transparency or color. The results demonstrated remarkable stability of using the material as a dosimeter [80].

#### 3.3.7. Thermal Fading

Thermal fading is defined as the loss of stored thermoluminescence TL intensity during the storage after irradiation. Typically, the thermal stability of the dosimetry material is based on two categories: annealing, storage temperature, readout, radiation types, and time, which are discussed in the previous terms. Second, the thermal stability is strongly dependent on the nature of the material; in this context, the activation *E* energy of the depth trap should be $\left(E>kT\right)$, to liberate an electron from a trapping center.

The desirable dosimeters possess a glow curve that starts upward after 140 °C and reaches a maximum peak between 200–250 °C. However, commercial TLD materials possess fading. The fading may occur through defects or be due to their recombination in trapped holes [81,82,83]. The thermal fading property of the new host TL material in this work was examined. In this case, 0.5 mol% Ag-doped ZnO was exposed to 1 Gy and 4 Gy. The irradiated TL materials were divided into two groups, where each group had 35 samples and was stored in an opaque box (dark environment) at room temperature. The readout of TL started directly after 1 h and continued for 45 days.

**Figure 15:**
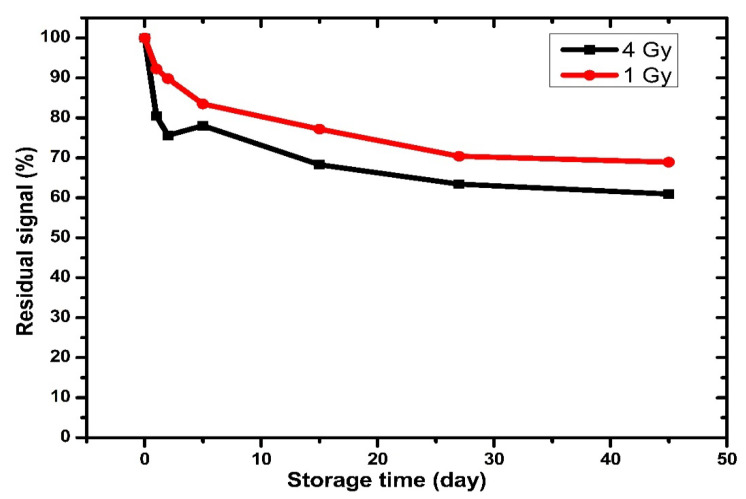
Thermal fading characteristics of Ag-doped ZnO thin films exposed to X-ray.

Figure 15 displays the fading curve behavior of the samples. The signal loss was recorded at 8% for 1 Gy and 20% for 4 Gy in the first hour. After 45 days of irradiation, the signal loss was recorded at 32% and 40% for the cases of 1 Gy and 4 Gy, respectively. The residual signal noticeably decreased with the increase in the dose value of the X-ray radiation.

#### 3.3.8. Optical Fading

Optical fading is another significant parameter for TLD materials; however, the optical fading dramatically depends on the light intensity, wavelength, and time exposure [83]. In the current study, optical fading was measured to estimate the sensitivity of the Ag-doped ZnO thin films to sunlight and room light. The samples were initially kept in a dark container during all the time measurements. Two groups of 30 samples were irradiated by 4 Gy of X-ray radiation and numbered, where each data point identified the outcomes of five samples. For six hours, the first group of samples was exposed to sunlight, while the second group was exposed to room light (by fluorescent lamp).

**Figure 16:**
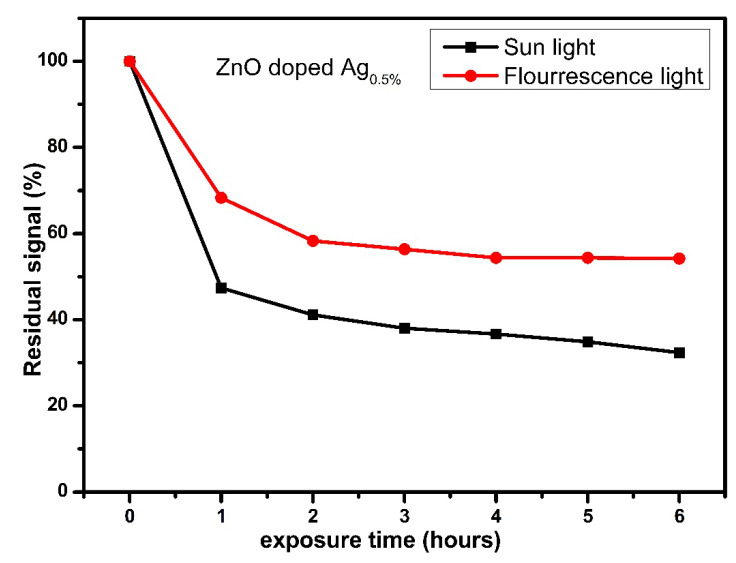
The optical fading of Ag-doped ZnO thin films exposed to sunlight and fluorescent light.

Figure 16 shows the behavior of the optical fading of Ag-doped ZnO thin films. The stored signals for the first group of samples yielded a loss of 53% after 1 h and 70% after 6 h of direct sunlight exposure in ambient conditions. As for the second group of samples, the stored signals yielded a loss of 30% after 1 h and 46% after 6 h. The trapped electrons or holes can be released optically at low temperatures, which suggests that further recombination could occur between the opposite charge carriers with an increasing absorbed temperature. Thus, a decrease in the TL intensity is expected when the proposed dosimeters are directly exposed to sunlight or room light [84]. This behavior has been observed in our previous works with multilayer thin films and nanopowder (pellets). The results revealed that the samples exposed to sunlight lost the signal more than the samples exposed to room light; this shrinking in the stored signals is attributed to the UV in sunlight. In light of this, the study concluded that the TL dosimeters should be stored in opaque containers when utilized [25].

#### 3.3.9. Minimum Detectable Dose (MDD)

For this study, the minimum detectable dose (MDD), also known as the lowest level detection, was calculated using Equation (6):

(6) $$D_{0}=\left(B^{*}+2\sigma B\right)F$$

where *σB* is the standard deviation of the background; B* is the average background TL (zero dose reading); and F is the calibration factor expressed in Gy nC^−1^.

In the current study, the five samples were read to measure the background before irradiation and exposed to 1 Gy of X-ray radiation. The average background and standard deviation for Ag-doped ZnO thin films were 0.414 and 0.099 nC, respectively. Additionally, the calibration factor recorded 0.0168 Gy nC^−1^, substituting Equation (7). Consequently, the low detectable dose of the Ag-doped ZnO thin film was found to be 10.31 mGy. Table 2 shows the MMD for the Ag-doped ZnO thin film. The type of model of TLD reader is a significant factor in determining MMD [85].

(7) $$F=Dose\;\left(Gy\right)/TL\;\left(nC\right)$$

#### 3.3.10. Percentage Depth Doses (PDD)

The percentage depth dose (PDD) is vital to determine the dose delivery, especially for cases that involve applying a narrow beam and a small field size [86]. PDD is given by the division of the absorbed dose at any depth, D, to the absorbed dose at a specific reference dose, which is along the central axis of the beam, as shown in Equation (8):

(8) $$\%PDD=D_{d}/D_{0}$$

In this study, X-ray radiation (80 kVp, 100 Ams) was applied to determine the depth dose distribution and compare the use of a PTW Markus parallel plate chamber, TLD rods, and TLD 100 chips, a host material of Ag-doped ZnO thin films. As previously described in Figure 1, with a slight difference, the procedure was set up with a source-to-surface distance (SSD) of 80 cm and a field size of 20 × 20 cm^2^. Furthermore, the phantom consisting of Perspex slabs included a slab with a thickness of 1 cm, where 10 slabs were used to get a maximum depth of 10 cm. The thin film set up in a slab with a thickness of 2 mm was placed in square holes, and the dose delivery was 3 Gy. The ionizing chamber, TLD rods, TLD 100 chips, and Ag-doped ZnO thin films were placed at different depths (from 0 to 10 cm).

**Figure 17:**
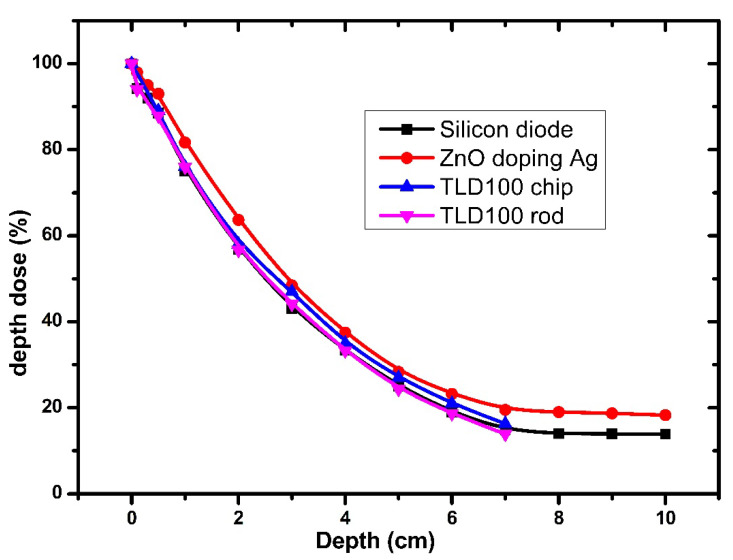
Percentage depth dose curve of the Perspex phantoms in 30 cm × 30 cm field size at 60 kVp of the X-ray energy with delivery dose 3 Gy.

Figure 17 shows the performance of the measurements at different depths, from the surface *D*_0_ up to *D* = 10 cm. The depth doses (each data point representing five samples’ outcomes) were normalized to *D*_0_ = *D_max_*. Ag-doped ZnO and all references recorded *D_max_* at a depth of 0 cm. The *PDD* appeared to gradually decrease from the surface until the depth of 6 cm. The *PDD* values of Ag-doped ZnO at a depth of 5 cm were higher than the *PPD* values of the TLD 100 chips, ionization chamber (IC), and TLD rods, which were found to be 28.33%, 24.33%, 27.00%, and 25.00%, respectively. The variation in the values of depth dose is subjected to many factors, such as the location of effective point samples, field size (may be due to the scattering of radiation), measurement of detectors, presence of air gap between detectors and layers of Perspex phantom, and the Z*_eff_* of the materials.

## 4. Conclusions

The proposed dosimeter thin films were prepared successfully by the hydrothermal (autoclave) method. The structural and optical properties were studied. The PL spectra showed two peaks that included the near bandgap edge emission (3.26 eV) and high-intensity defect in the visible region with a wide range (2.25 to 1.55 eV). The dosimetric properties were investigated. TL optimum intensity was found at 0.5 mol% of Ag for a delivered dose of 4 Gy, with the glow curve showing a single peak at 270 °C. The TL sensitivity was normalized to the mass of the thin films and found to be two times that of the TLD 100 chips. Ag-doped ZnO thin films showed good reproducibility, and the results of PDD also confirmed an agreement with the case of TLD 100. However, it can be concluded that the advantage is that the glow curve’s growth was acquired immediately with different heating rates and that the sensitivity is more than that of TLD 100. The limitations of this work are that the thin films’ synthesizing conditions were a crucial effect on the shape and position of the glow curve and optical fading. These thin films were strongly affected by sunlight. The study demonstrated that the synthesized sample exhibits suitable TL properties for radiation monitoring.

## Figures and Tables

**Table 1:** Calculation of the crystallite size of ZnO and Ag-doped ZnO thin films.

Samples	2 θ (°)	FWHM (rad)	Crystallite Size (nm)
Pure ZnO	31.80	0.147	36.21
1% Ag	31.79	0.1476	35.9
3% Ag	31.52	0.197	27.0
5% Ag	31.76	0.246	21.5

**Table 2:** The minimum detectable doses (MMD) for the Ag-doped ZnO thin film.

1 Gy X-ray Irradiated	Ag-Doped ZnO		
Samples	BG nC	TL Signal nC	TL–BG
1	0.27	63.27	63
2	0.36	59.62	59.26
3	0.47	48.94	48.47
4	0.52	65.28	64.76
5	0.451	61.85	61.399
Average	0.4142	59.792	59.3778
STDV	0.099238		
		F	0.016841311
		D_0_	0.01031827

## Data Availability

The data presented in this study are available on request from the corresponding author.
